# Effects of Hyperbranched Polyethylenimine on the Properties of Commercial Epoxy Resins for Composite Applications

**DOI:** 10.1021/acspolymersau.5c00141

**Published:** 2025-12-02

**Authors:** Zois Tsinas, Ajay Krishnamurthy, Qi An, Ran Tao, Amanda L. Forster, Aaron M. Forster

**Affiliations:** † Theiss Research, La Jolla, California 92037, United States; ‡ Material Measurement Laboratory, 10833 National Institute of Standards and Technology, Gaithersburg, Maryland 20899, United States; § Department of Chemical and Biomolecular Engineering, North Carolina State University, Raleigh, North Carolina 27695, United States

**Keywords:** epoxy, matrix, blends, hyperbranched amine, composites, cure kinetics

## Abstract

Hyperbranched polymers are commonly used as surface and cure modifiers, along with nanoparticles, of epoxy resins used in medical and infrastructure applications; however, their impact on structure and performance of the epoxy resins is not fully understood. Here, a commercial epoxy-aromatic amine resin (EP-AA) system is blended with off-stoichiometric amounts of hyperbranched amine (HA), and the cure kinetics, structure, polymer dynamics, and mechanical properties of the different compositions are evaluated. Cure kinetic studies indicate that the curing of the hyperbranched amine is nearly complete prior to that of the aromatic amines. At its highest concentration (1% by mass), the hyperbranched amine is found to autocatalyze the epoxy and the aromatic amine cure, but the bulky HA groups slow the diffusion kinetics during the final stages of the cure process. This results in a heterogeneous, cocontinuous network structure consisting of unreacted secondary amines that increase the side-group/pendant contribution and increase the free volume within the polymer network. The restricted cooperative segmental mobility of the polymer network in the 1% by mass HA blend results in poor energy dissipative properties, as evidenced by reduced strength and strain to failure.

## Graphical abstract

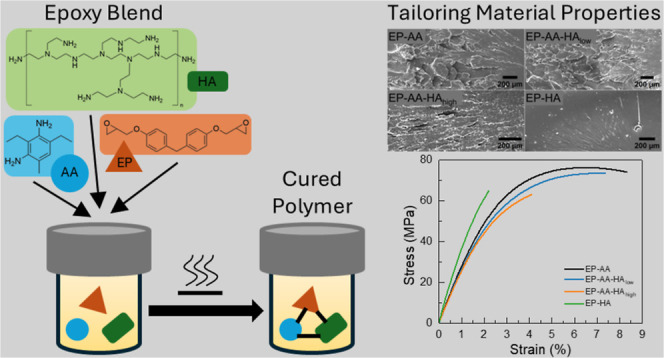

## 1 Introduction

Hyperbranched polymers (HBPs), such as hyperbranched polyethylenimine (h-PEI), are dendritic structures that are increasingly utilized for various applications such as gene therapy,^1^ drug delivery,^2^ gas sensing,^3^ and heavy metal removal.^4^ A typical dendritic structure is highly globular and consists of a core of repetitive units that terminate in a periphery of functional sites. A hyperbranched polymer (HBP) is an imperfect counterpart that retains some of this globular structure along with an irregular distribution of terminal groups, dendritic groups, and linear functionalities.^5^ Achieving a purely dendritic structure requires a complex, well-controlled, multistep procedure, whereas a simple, one-step polymerization process can synthesize a hyperbranched structure. The polydispersity of a hyperbranched structure allows for a lower cost of production compared to uniform dendritic architectures,^6^ while a large number of end groups improve their functionality over linear polymers.^7^ Furthermore, comparing HBPs and linear polymers of similar molar masses, the large degree of branching in HBPs results in a lower degree of chain entanglement, which allows for easier mixing with solvents and other polymers.^7^ These desirable properties have made HBP an attractive choice that offers a compromise between purely dendritic structures and linear polymers. It has also enabled their use as additives and modifiers in traditional epoxy-based matrices.

In general, epoxy materials are a widely used commodity in various applications, including coatings, structural adhesives, and electronics due to their excellent scratch resistance, superior stiffness compared to other engineering polymers, temperature stability, and chemical resistance. However, epoxy materials inherently suffer from high brittleness that can often limit their applicability. Dendritic polymers and HBPs have been utilized to toughen epoxy resin systems and are either implemented as additives that phase separate during the curing process^8–12^ or as a complete replacement to their aliphatic or aromatic analogs.^13,14^ The majority of these studies employ a controlled phase separation strategy where, at a given composition of the blend, a homogeneous mixture is tailored to undergo phase separation during cure by manipulating the temperature, chemical reactivity, structural topology, and molar mass of the HBPs. These phase-separated domains blunt crack growth and improve the toughness of epoxy matrices. Depending on the structure of the HBP, other polymer properties, such as glass-transition temperature and moduli (e.g., Young’s modulus, storage modulus), can be modified. Early studies examining the use of HBPs in epoxy matrices were largely focused on polyester,^8–10^ but later work explored other variations such as epoxide^15–17^ and poly­(amino-ester)­s or poly­(ester-amide) that have hydroxyl-terminated structures.^18,19^

Recently, the use of h-PEI in structural applications has gained interest within the scientific community. Although h-PEI has been explored as an alternative to traditional aliphatic amines,^14,20^ they are primarily used to manipulate the surfaces of nanomaterials.^21,22^ H-PEIs are a water-soluble, weak base that can be easily protonated and used to create stable suspensions of nanoparticles in polar media. In addition, the amine sites can covalently react with an epoxy matrix and improve load transfer between the nanoparticles and the matrix, as observed previously in h-PEI-modified graphene oxide,^23^ carbon nanotubes (CNTs),^24,25^ silicon dioxide (SiO_2_),^26^ and clay^27^-based composites. The challenge, however, is to understand the contributions of HA since their behavior is so strongly coupled with the nanoparticles that it is difficult to separate the contribution of HA alone. This phenomenon was demonstrated in our previous study, where the presence of only 1.8% by mass CNT-(h-PEI) (1.4% CNTs and 0.4% h-PEI) affected the polymer dynamics and provided hierarchical structural reinforcements at multiple length and time scales within a fiber-reinforced polymer composite.^28^ Although it is often expected that the effect of small mass fractions of polymer modifiers would be minimal, studies on utilizing h-PEI as a replacement for aliphatic and aromatic amine curing agents reveal that the interactions between hyperbranched amine (HA) and an epoxy resin (EP) matrix can be complex and are related to the molar mass and degree of branching of HA used.^7,14,29^

Santiago et al. studied the differences in the curing process between h-PEI (Mw ≈ 2000 g/mol) and aliphatic triamine hardener in a difunctional epoxy matrix.^14^ The highly functional h-PEI slows the kinetics of the EP-HA curing process and increases the cross-link density, as compared to the aliphatic triamine hardener. In their subsequent study, the authors also compare this behavior with a lower molar mass h-PEI (Mw ≈ 800 g/mol) against the same aliphatic diamine hardener, where topological constraints introduced in HA architecture were found to introduce incomplete cure in the final product.^29,30^ Another study by the same authors involved comparing a difunctional epoxy matrix cured with aliphatic diamine hardener and h-PEI of different molar masses (approximately 800 g/mol and 2000 g/mol) in the presence of an anionic initiator.^31^ Here, it was observed that increasing the molar mass of the aliphatic diamine from about 800 to 2000 g/mol increased the mobility restrictions and permanent chain entanglements in the polymer network. As a result, the glass-transition temperature was affected and the toughness and ductility of the matrix were reduced. Studies by Román et al. comparing properties of h-PEI with different molar masses (approximately 2000 g/mol and 25,000 g/mol) and difunctional epoxy mixtures indicate lower amine reactivity as molar mass increased, which results in lower exothermic heat of reaction from the epoxy-amine cure process.^7,13^

In this work, we evaluate the effect of adding HA on the overall properties of an aromatic amine and epoxy matrix in the absence of elastic reinforcements such as fibers and nanoparticles. Small mass fractions (%) with nominal values of 0.4 and 1 of h-PEI (Mw ≈ 25,000 g/mol) are mixed with off-stoichiometric reaction mixtures of difunctional aromatic epoxy and aromatic diamine resin system to create epoxy-amine compositions with excess amine. Their properties are then compared with those of stoichiometric epoxy-aromatic amine and epoxy-hyperbranched amine controls. The curing process is monitored using differential scanning calorimetry (DSC) measurements, and model-free isoconversional analysis was used to understand changes in the cure kinetics due to HA addition. The viscoelastic and free volume characteristics of the isothermally cured samples are investigated by using dynamic mechanical thermal analysis (DMTA) and positron annihilation lifetime spectroscopy (PALS). The data obtained are then compared to understand the differences in the polymer network architecture introduced by HA addition. To understand the practical application of HA addition, the tensile properties of the blends were evaluated, and the failed cross sections were imaged using a scanning electron microscope (SEM). It is observed that even at small off-stoichiometric compositions, the large molar mass (about 25,000 g/mol), highly branched h-PEI affects the polymer network heterogeneity in the blends and leads to significant changes in their mechanical properties, which could potentially reduce the effectiveness of nanoparticle reinforcements in structural applications.

## 2 Materials and Methods

### 2.1 Materials

Epoxy specimens were prepared using a one-to-one stoichiometric mixture (mix ratio of nominally 100/26.4 by mass) of bisphenol F epichlorohydrin epoxy monomer (Epon862; Hexion Specialty Chemicals[Fn fn1]) and diethyl toluene diamine (aromatic amine) curing agent (Epikure W Curing Agent; Hexion Specialty Chemicals). These samples are noted in this study as EP-AA (two component mixture). Also, samples with bisphenol F epoxy monomer and an aliphatic amine as curing agent, hyperbranched polyethylenimine (polyethylenimine, branched, *M*_w_ ≈ 25,000; Sigma-Aldrich), were prepared at a similar one-to-one stoichiometric mixture with a mix ratio of nominally 100/18.6 by mass. These samples are noted herein as EP-HA and did not contain any of the aromatic amine molecules in the mixture (two component mixture). Finally, mixtures of all three components were prepared that contained both the aromatic amine and hyperbranched polyethylenimine as curing agents at nominal mix ratios of 100/26.4/0.5 (EP-AA-HA_low_) and 100/26.4/1.26 (EP-AA-HA_high_) by mass, respectively. For all the isothermally cured samples tested in this study, the liquid constituents were hand mixed and degassed at about 40 °C under vacuum for approximately 10 min. Then, individual mixtures of EP-AA, EP-AA-HA_low_, and EP-AA-HA_high_ were isothermally precured in a convection oven at around 130 °C for approximately 6 h and postcured in a vacuum oven at around 150 °C for about 24 h. In the case of EP-HA mixtures, they were initially precured in a convection oven at around 90 °C for about 6 h and postcuring was performed in a vacuum oven at approximately 120 °C for around 24 h. Finally, for the dynamically cured samples, the degassed mixture was transferred into differential scanning calorimeter (DSC) pans and immediately placed in the DSC cell to dynamically cure according to the selected protocol, as described in the section below.

### 2.2 Calorimetry and Dynamic Curing of Epoxy Blends

DSC was used for the dynamic curing of samples and evaluation of their thermal properties during cure as well as after cure in the case of isothermally cured samples in a convection oven. Isothermally cured epoxy samples with masses between 5 and 10 mg were placed in hermetically sealed aluminum pans. Samples containing only HA (EP-HA) were heated from around −60 to 189 °C and the rest of the samples (EP-AA, EP-AA-HA_low_, and EP-AA-HA_high_) were heated at temperatures from around −60 to 325 °C. The heating rate was kept constant across all the measurements and set at 10 °C/min. Similar sample masses were used for the dynamically cured specimens, and the cure kinetics study was completed at the same range of temperatures but at various heating rates. For the mixtures of EP-AA, EP-AA-HA_low_, and EP-AA-HA_high_, heating rates of (2, 4, 5, 8, and 10) °C/min were used, while for the EP-HA mixtures, the heating rates used were (2, 5, 10, 15, and 20) °C/min.

### 2.3 Viscoelastic Characterization

DMTA measurements were conducted on the specimens using an ARES-G2 rheometer equipped with a stainless-steel rectangular torsion fixture and a liquid–nitrogen-cooled forced-convection oven. Rectangular specimens with nominal dimensions of 33 mm in length by 12 mm in width by 1.5 mm in thickness were used. The rheometer was initially calibrated according to the manufacturer specifications, and standard uncertainties associated with the frequency and modulus responses were within ±5% and ±8%, respectively. Tests were configured to automatically adjust strain and axial load levels to remain within the linear viscoelastic range of the epoxy samples and greater than the operational noise floors of the instrument. For temperature measurements, a standard uncertainty of ±0.1 °C was assumed. Temperature sweeps (2 per sample) were conducted between 189 °C and −150 °C and were normalized and graphed for each epoxy blend based on each sample’s alpha relaxation temperature (*T*_α_) for comparison (*T*_α_ + 40 °C and *T*_α_ – 300 °C). An oscillating frequency of nominally 1 Hz was used, and a cooling rate was set at 1 °C/min. Prior to the sweep, all samples were held at nominally 189 °C for about 1 h to remove the thermal history. Strain sweeps were performed prior to the experiments to confirm that the initially set strain percentages at 189 °C were within the linear viscoelastic range with sufficient signal/noise in the torque signal (minimum 200 μN·m) greater than *T_α_*. The set strain values were automatically controlled (reduced) at each temperature step by the instrument to stay within a specified torque limit (200 to 500 μN·m).

### 2.4 Free Volume Analysis

PALS is an electron–positron annihilation-based measurement that is often utilized to understand free-volume characteristics in polymeric materials. In materials with low electron densities, such as polymers, thermalized positrons form a metastable bound state with an electron, termed a positronium, which is largely confined to the pore structures/free volume.

Isothermally cured samples with approximate dimensions of 25 mm × 25 mm × 1.5 mm were tightly clamped on either side of a positron emission source “sandwiched” between glass slides. The source consists of ^22^NaCl salt (activity: 10 μCi) placed between two Kapton foils that are ≈50 μm thick and sealed using a commercial adhesive. The sample–source–sample assembly was wrapped in aluminum foil and placed between two plastic scintillators that are each associated with a photomultiplier tube (PMT). The scintillator/PMT pair was designated to detect the γ-ray signatures corresponding to the positron emission process (≈1.27 MeV) and the subsequent positron/positronium (Ps) annihilation processes (≈0.511 MeV). The time difference in the positron production and the annihilation within the sample is recorded to generate the positron annihilation lifetime spectrum, which was analyzed using commercially available software. For each specimen, nearly eight million counts of annihilation data were collected under room-temperature conditions and atmospheric pressure.

### 2.5 Evaluation of Mechanical Properties

The mechanical properties of the epoxy blends studied herein were measured on a universal load frame Instron/MTS instrument equipped with a 500 N load cell. The polymer samples were cast and cured into dog-bone shaped specimens with nominal dimensions of 165 mm in length, 19 mm in width, and 3 mm in thickness, based on the isothermal curing method described in Section 2.1 above. In addition, the narrow section of the samples had a nominal width of 13 mm and a length of 57 mm. Testing was performed at a constant strain rate set at 0.06 s^–1^. The stress versus strain behavior of each epoxy blend was tested, and the ultimate tensile strength (UTS), strain to failure (ε_f_), and Young’s modulus (*E*) were evaluated.

### 2.6 Surface Morphology

The morphology of the cross-sectional area of the tensile tested samples at the fracture site was evaluated via SEM, on an FEI Helios NanoLab 650 dual-beam SEM. Specimens were mounted on aluminum stubs with carbon conductive tape and sputter coated with nominally 4 nm of gold–palladium (Au–Pd) conductive coating for optimal imaging conditions. The micrographs of the specimens were collected under high vacuum, i.e., less than about 0.4 mPa (3 × 10^–6^ Torr), at a beam energy of around 5 keV and probe current between 0.1 pA and 0.5 pA, with scan parameters optimized for clear images with minimal charging, artifacts, and drift.

## 3 Results and Discussion

### 3.1 Chemical Structure and Reaction Mechanisms

The general structures for the epoxy, aromatic amine, and hyperbranched amine are given in Figure 1.

**Figure 1:**
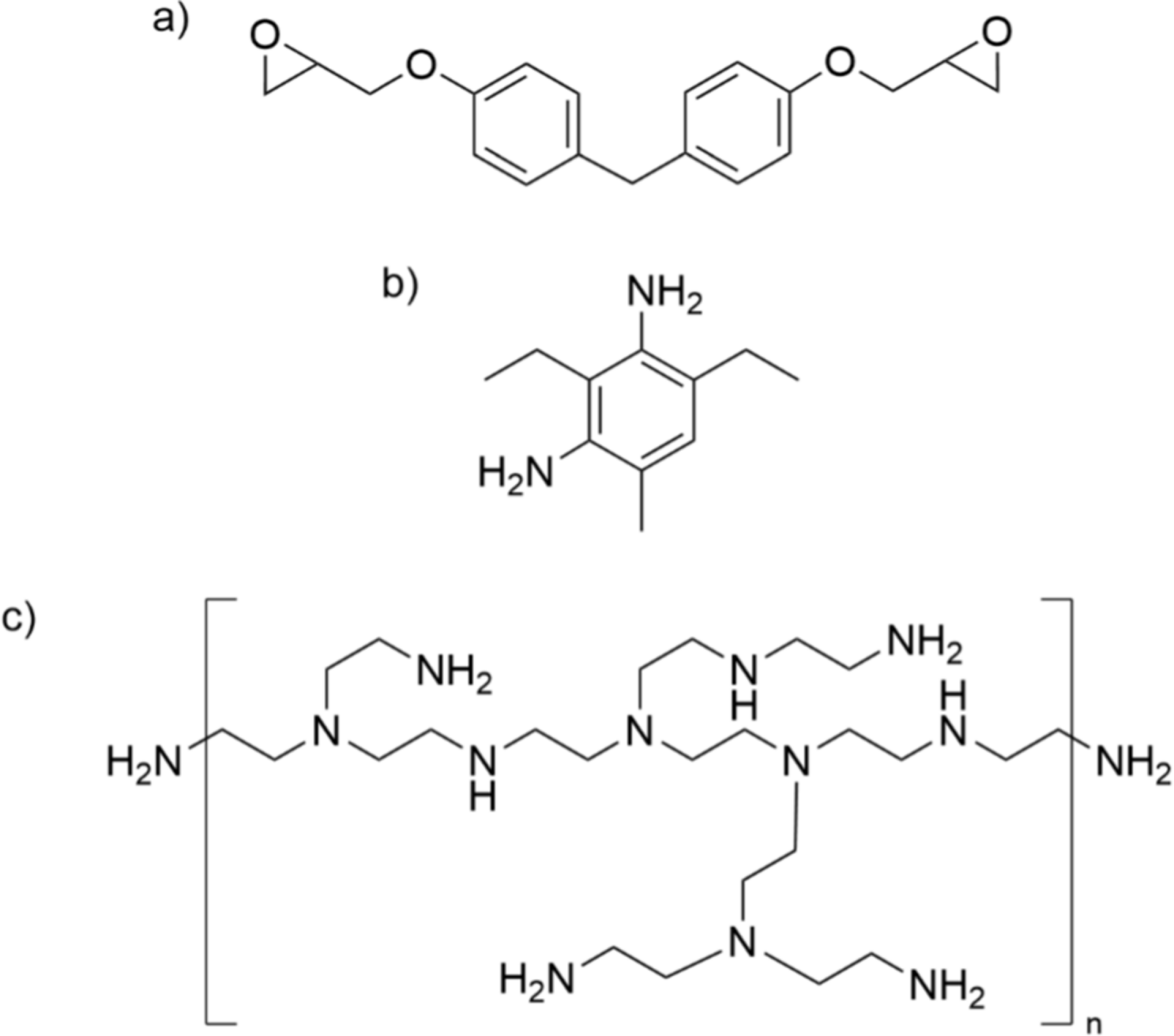
Chemical structure of (a) bisphenol F (DGEBF), (b) diethyl toluene diamine (DETDA)—aromatic amine, and (c) hyperbranched polyethylenimine—aliphatic amine used in this study.

The manufacturer’s recommendation for the epoxy-aromatic amine mix ratio (EP-AA) is based on their corresponding equivalent masses of epoxide and amines, respectively. Those values of epoxy equivalent weight and amine hydrogen equivalent weight (AHEW) are given by the manufacturer, and the recommended mix ratio is nominally 100/26.4, as shown in Table 1. The addition of HA to the composition, as a third component, will lead to a slight excess of overall amine concentration in the triblend systems, as shown in Table 1. Based on the mass fraction of these triblend systems, a stoichiometric ratio between epoxide to amine groups was calculated and is presented in Table 1. Finally, the epoxy-hyperbranched amine mix ratio (EP-HA) was estimated based on a one-to-one stoichiometric ratio and is presented in Table 1. A number for average active hydrogen atoms per nitrogen atom of one was assumed, and the molar mass of the polymer given by the manufacturer was 25,000 g/mol. These numbers were used to determine a theoretical value of the AHEW for the branched amine molecule used in this study. Also, we expect that only the hydrogens found on the primary (NH_2_) and secondary (NH) amine groups can participate in reactions with the epoxy resin. Tertiary amines do not have any hydrogens and are not expected to affect the curing process. Similar off-stoichiometric compositions in the triblend systems are not expected to result in significant changes to the overall properties, but the architecture of the HA is shown to have a more detrimental influence on the properties, as discussed in a later section.

**Table 1:** Specimens Used in the Study and Their Percent by Total Mass of HA Added to the Three-Component Blends, Nominal Composition Ratios by Mass of the Various Components Used, and Stoichiometric Ratios

specimen	% by mass of HA (%)	total composition (EP/AA/HA)	stoichiometry (eq of epoxide/eq of amines)
EP-AA	-	100/26.4/0	1:1
EP-AA-HA_low_	0.4	100/26.4/0.5	1:1.02
EP-AA-HA_high_	1	100/26.4/1.26	1:1.06
EP-HA	-	100/0/18.6	1:1

In general, epoxy cured in equivalent or excess amine stoichiometry follows the simplified condensation reaction based on the rate-controlling primary and secondary amine reactions with epoxide groups, as shown in Scheme 1.

**Scheme 1:**
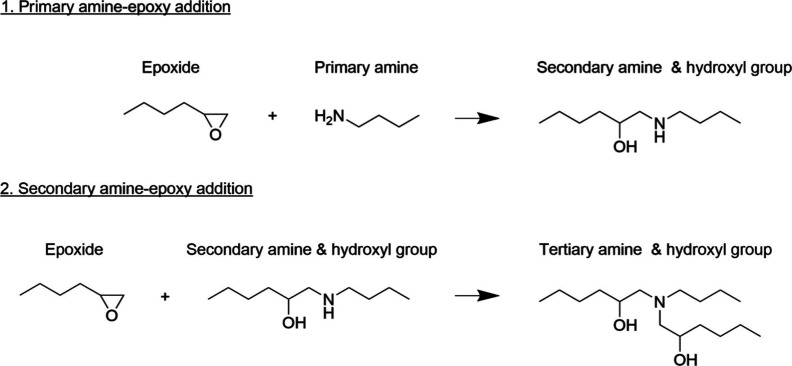
Typical Epoxy-Amine Condensation Reaction Mechanism of the Primary and Secondary Amine Groups with the Epoxide Group

### 3.2 Dynamic Cure Behavior

The simplified reaction mechanism shown in Scheme 1 is considered valid for EP-AA and EP-HA controls; however, the reaction mechanism in the blends studied herein is anticipated to be more complex because of the differences in the curing kinetics between hyperbranched aliphatic and aromatic amines. To highlight the differences across samples with varying compositions and stoichiometries, heat flow as a function of temperature at a single heating rate (set at 10 °C/min) is shown in Figure 2.

**Figure 2:**
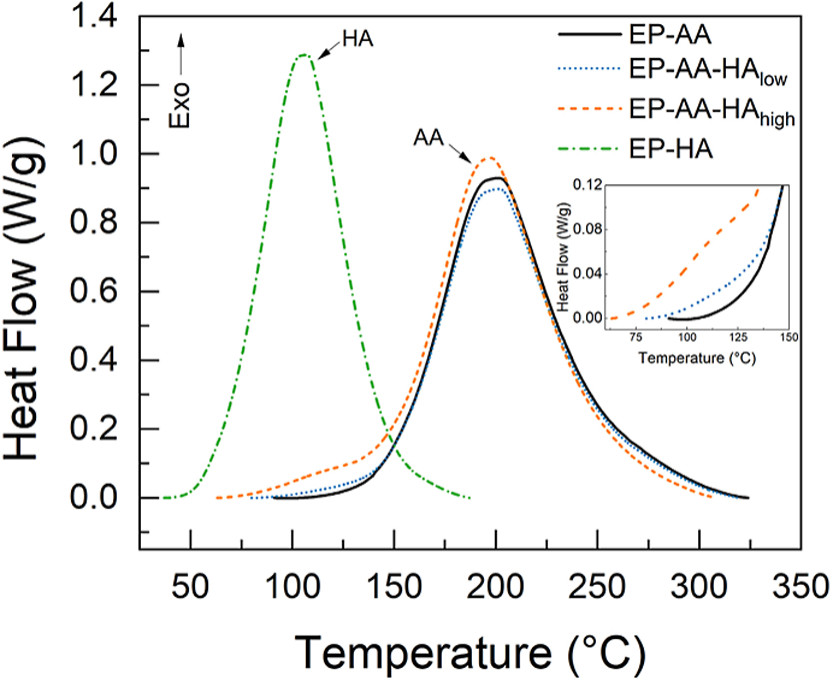
DSC thermograms on the dynamic curing reactions of the various epoxy blends. Standard uncertainties associated with the use of DSC are approximately ±5%.

The heat flow data from the thermograms shown in Figure 2 support that the nucleophilic substitution reaction between EP and AA (shown in Scheme 1) starts at higher temperatures, and the curing process occurs over a larger temperature range compared to that of the EP-HA. This is attributed to the electron-withdrawing nature of the aromatic amines and the steric hindrance from their aromatic chemical structures. In contrast, the highly reactive aliphatic amines can readily undergo nucleophilic substitution at lower temperatures.^32^

Adding HA to the EP-AA blend can effectively shift the onset of the epoxy-amine cure reaction to lower temperatures. As the mass fraction of HA increases from about 0.4% to 1%, the onset temperature of the curing process approaches that of the EP-HA control (Figure 2). It is interesting to observe such a notable shift in the cure onset of the blends, even at such low mass fractions of HA. In the EP-AA-HA_low_ composition, the HA curing appears as a subtle increase in the heat flow response between 80 and 120 °C (as highlighted in the inset of Figure 2). A more defined HA curing shoulder appears between 60 and 125 °C in the EP-AA-HA_high_ composition than for the EP AA HA_low_ composition, which abruptly transitions to higher temperatures during the curing of EP-AA blends. The presence of these low-intensity shoulder peaks at lower temperatures indicates that a portion of the EP-HA curing may be completed before the commencement of the EP-AA cure.

The enthalpy of the reaction and glass-transition temperature for all dynamically cured epoxy blend samples at a heating rate of 10 °C/min are given in Table 2. It has been previously observed that the presence of larger molar mass species with branched architecture may introduce diffusion limitations in the curing process and can result in lower Δ*H* in the EP-HA specimen when compared to the EP-AA specimen, as shown in Table 2.^7,33^ This could potentially be explained as higher molar mass HA forms tight covalent networks that impose mobility restrictions. These topological constraints, as the curing process takes place over time, can create domains within the material with higher concentrations of epoxy and others with higher concentrations of PEI, resulting in lower amine reactivity and possibly an incomplete cure. Finally, an increase in the enthalpy of the reaction was observed in the EP-AA-HA_high_ composition when compared to the control EP-AA and EP-AA-HA_low_ blends. This can possibly be due to the excess of amine contents in this blend since the stoichiometric ratio between epoxy and amine is 1:1.06 which is the highest of all the different blends used in this study.

**Table 2:** Enthalpy (Δ*H*) of Reaction and Glass-Transition Temperature (*T*_g_) for All Samples from the Dynamic Cure Study in the DSC at a Heating Rate of 10 °C/min^a^

sample	Δ*H* (J/g)	*T*_ge_ (*T*_gp_^*^) (°C)
EP-AA	407.7	142.7
EP-AA-HA_low_	397.6	143.7 (147.4*)
EP-AA-HA_high_	436.1	137.2 (146.8*)
EP-HA	392.6	46.2, 85.6

a *T*_ge_ are the experimental values and *T*_gp_ are the values denoted with an asterisk* and indicate prediction using eq 1. Uncertainties for the enthalpy and temperature data reported are within a ±5% error based on the manufacturer’s specifications. The EP-HA sample revealed two distinct values of *T*_ge_ separated in the table with a comma.

A second DSC heat scan on the dynamically cured samples revealed different glass-transition behaviors between the examined blends (data not shown here for brevity). Specifically, the EP-HA material exhibit two distinct *T*_g_ values, while the other materials showed a single *T*_g_. The measured calorimetric *T*_g_ values (*T*_ge_) of the blends are reported in Table 2. This could be explained by the nonuniform cross-link density in this blend creating regions with higher cross-linking, revealing a higher *T*_g_, and lower cross-linking, which results in a lower *T*_g_, respectively. Knowing that the EP-HA blend was mixed to hold a 1:1 stoichiometric ratio of epoxy/amine, it is more likely that the presence of two distinct *T*_g_ values could be related to the reaction kinetics and the diffusion limitation of the larger molar mass and at the same time branched HA molecule in the blend. These inhomogeneities in the cross-link density can potentially lead to different segmental mobility within the unevenly cured epoxy network and phase separation, which in turn translates as two glass-transition temperatures. A similar behavior revealing not a sharp but a broad *T*_g_ profile has been observed in difunctional epoxy blends with lower h-PEI molar mass (about 2000 g/mol) in a previous study.^7^ Finally, there is no significant change in the *T*_g_ values obtained from the EP-AA and the EP-AA-HA_low_ or EP-AA-HA_high_ blends.

Despite its applicability to thermoplastic blends, the *T*_g_ changes in the blend compositions are modeled using the Fox equation^34^ (eq 1), and the outcomes are shown within parentheses in Table 2.

1 $$\frac{1}{T_{gp}}=\frac{w_{a}}{T_{ga}}+\frac{w_{b}}{T_{gb}}$$

where *T*_gp_ is the predicted glass-transition temperature of the blend; *w*_a_, *w*_b_, *T*_ga_, and *T*_gb_ are the mass fractions and glass-transition temperatures of the various components (*w*_a_ denotes EP-AA and *w*_b_ denotes EP-HA). A good agreement, with a difference of 2.5% for the HA_low_ blend and 6.8% in the case of HA_high_ blend is found between the Fox model prediction and the experimental *T*_g_ values determined for these blends, which suggests homogeneous interactions between these entities, as shown in Table 2.

Reaction models are often used to better understand the kinetics of the curing process. In the case of blends, deconvolution of peaks with or without simultaneous fitting processes is often employed to varying degrees of success,^35–37^ but this approach is complicated by the magnitude of the PEI curing peak. Therefore, a model-free, isoconversional analysis approach is adopted here to showcase the general trends in reaction activation energies as a function of cross-linking.^38^ Isoconversional methods assume that the reaction rate at a particular extent of conversion of reactants to products is a function of only the temperature. Since the heat of cure at each dynamic ramp rate does not show systematic dependence on the heating rate,^39^ a differential method proposed by Friedman was utilized.^38,40,41^ The apparent activation energy of the cure reactions (*E*_a_) vs the degree of conversion (α) is shown in Figure 3.

**Figure 3:**
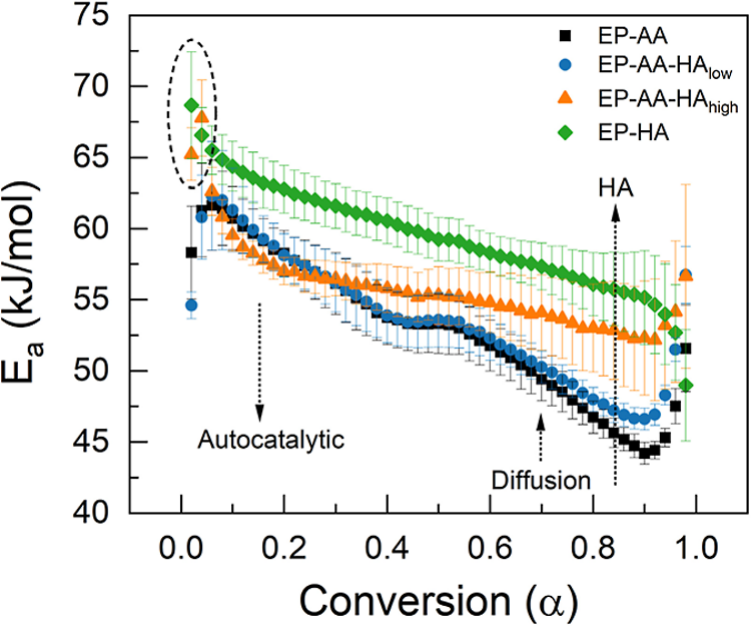
Dependence of apparent activation energy (*E*_a_) on the degree of conversion (α) for the different epoxy systems studied here. Data presented are mean ± standard error of the mean (S.E.M.), with *n* ≥ 3.

In the case of EP-AA control, the apparent activation energy (*E*_a_) for the entire process is around 55 kJ/mol, which is comparable to previous measurements on similar systems.^42^ Despite consisting of hyperbranched aliphatic amines, EP-HA exhibits a slightly greater apparent *E*_a_ (approximately 59 kJ/mol) than the EP-AA control, likely due to immiscibility between the aromatic and the hyperbranched amines. Also, the larger HA molar mass introduces greater diffusion limitations to epoxide reactivity compared to a lower molar mass molecule, as shown previously.^7^ Hence, despite having similar amine hydrogen equivalent weights, the EP-HA cure displays a slightly greater apparent *E*_a_ than does the EP-AA cure.

As shown in Figure 3, the apparent activation energy (*E*_a_) variation with respect to conversion (α) has a similar decreasing trend in the curing of the EP-AA and EP-HA controls. Based on prior literature, the curing process can be simplified to the following rate-limiting steps, as indicated in Scheme 1 above. Initially, the primary amine reaction with epoxide groups (α ≈ 0 to 0.2) that condense to form hydroxyl complexes and byproducts that catalyze subsequent chemical reactions with secondary amine sites (α ≈ 0.2 to 0.5).^14^ Then, once a cross-linked or a viscous network is formed (α > 0.5), the reaction kinetics shift from a chemically controlled process to a diffusion limited step that leads to a sudden increase in the apparent activation energies toward the end of these reactions (α > 0.8).^14^ The apparent *E*_a_ trends in the beginning (α < 0.1) and at the end (α > 0.9), as shown in Figure 3, are heavily influenced by the baseline used for data processing. However, since the HA reactions in this study are observed to reach completion within α < 0.1, these results can be used with confidence to discuss trends in the blends.

The observed values for the activation energy and the trends at various conversions display some differences in the blends compared to the EP-AA control. It is important to note that the apparent activation energies observed in this study can be interpreted as indicators of mechanism changes in the curing process rather than intrinsic activation barriers. The data suggest that the addition of a low concentration of HA does not significantly affect the cure kinetics except for a minor increase in the apparent *E*_a_ toward the end of the cure. However, adding a higher concentration of HA to the mixture resulted in obvious changes in the cure kinetics and the activation energy trend. A close examination of the apparent *E*_a_ values at the beginning of the curing process (low conversions) for the 1% HA mixture sample, reveals that the apparent *E*_a_ of the reaction between the epoxy and primary HA is similar to the EP-HA control, as shown in Figure 3 (indicated by a circle). This could be explained by considering that HA cures at lower temperatures than AA, and the majority of the HA cure is completed before the onset of AA cure (as shown in Figure 2). At around α ≈ 0.05, there is approximately a 10 kJ/mol decrease in the apparent *E*_a_ and a subsequent drop of about 2 to 3 kJ/mol at conversions between 0.05 and 0.2. This potentially signifies the commencement of the EP-AA cure catalyzed by the hydroxyl groups in the EP-HA cured material. Beyond a conversion of 0.3, the *E*_a_ trend no longer follows a similar profile to the ones observed in the EP-AA or EP-HA control samples and is found to reach a near steady-state value at around 55 kJ/mol. A similar trend for the activation energies has been observed by Sbirrazzuoli et al.,^43^ in the case of excess amine stoichiometry, that has been attributed to additional primary amines that preferentially have higher reactivity over secondary amines, although the amine concentration used in that study is off stoichiometry by 5-fold. In this study, the activation energy trend of the EP-AA-HA_high_ composition may be attributed to a combination of increased primary amine concentration due to minor amine off-stoichiometry and the diffusion barrier against the EP-AA curing reaction introduced by the presence of the already cured EP-HA network.

### 3.3 Viscoelastic Characterization of Isothermally Cured Specimens

Following the DSC dynamic cure experiments, larger specimens were cured isothermally according to the procedures outlined in the Materials and Methods section. These specimens were characterized via DMTA to understand viscoelastic properties using a temperature scan at a cooling rate set to 1 °C/min, a constant frequency (1 Hz), and a temperature range between about *T*_α_ + 40 °C and *T*_α_ – 300 °C (where *T*_α_ is the α-relaxation temperature defined by the peak of the loss modulus (*G*″) data, values for each sample are shown in Table 3). Figure 4 compares the responses from all of the samples around the α-relaxation process (segmental relaxation).

**Table 3:** Viscoelastic Properties along with the Molar Mass between Cross-Links and Relaxation Temperatures of the Different Isothermally Cured Epoxy Specimens and Blends^a^

sample	*G*_re_^′^ (*G*_rc_^′^)	*M*_ce_ (*M*_ct_) (g/mol)	*T*_α_ (°C)	*T*_β_ (°C)	*T*_ge_ (*T*_gp_) (°C)
EP-AA	11.6	427 (345–516)	154	–85	142.7
EP-AA-HA_low_	15.5 (11.65)	320*	154	–82	143.7 (153.4)
EP-AA-HA_high_	14.9 (11.78)	334*	148	–79	137.22 (153.1)
EP-HA	33.4	149* (245–368)	80 and 119	–60	46.2, 85.6

a The standard uncertainty on the frequency and the modulus responses were found to be within ±5% and ±8%, respectively. For temperature measurements, a standard uncertainty of ±0.1 °C is assumed based on manufacturer specifications.

**Figure 4:**
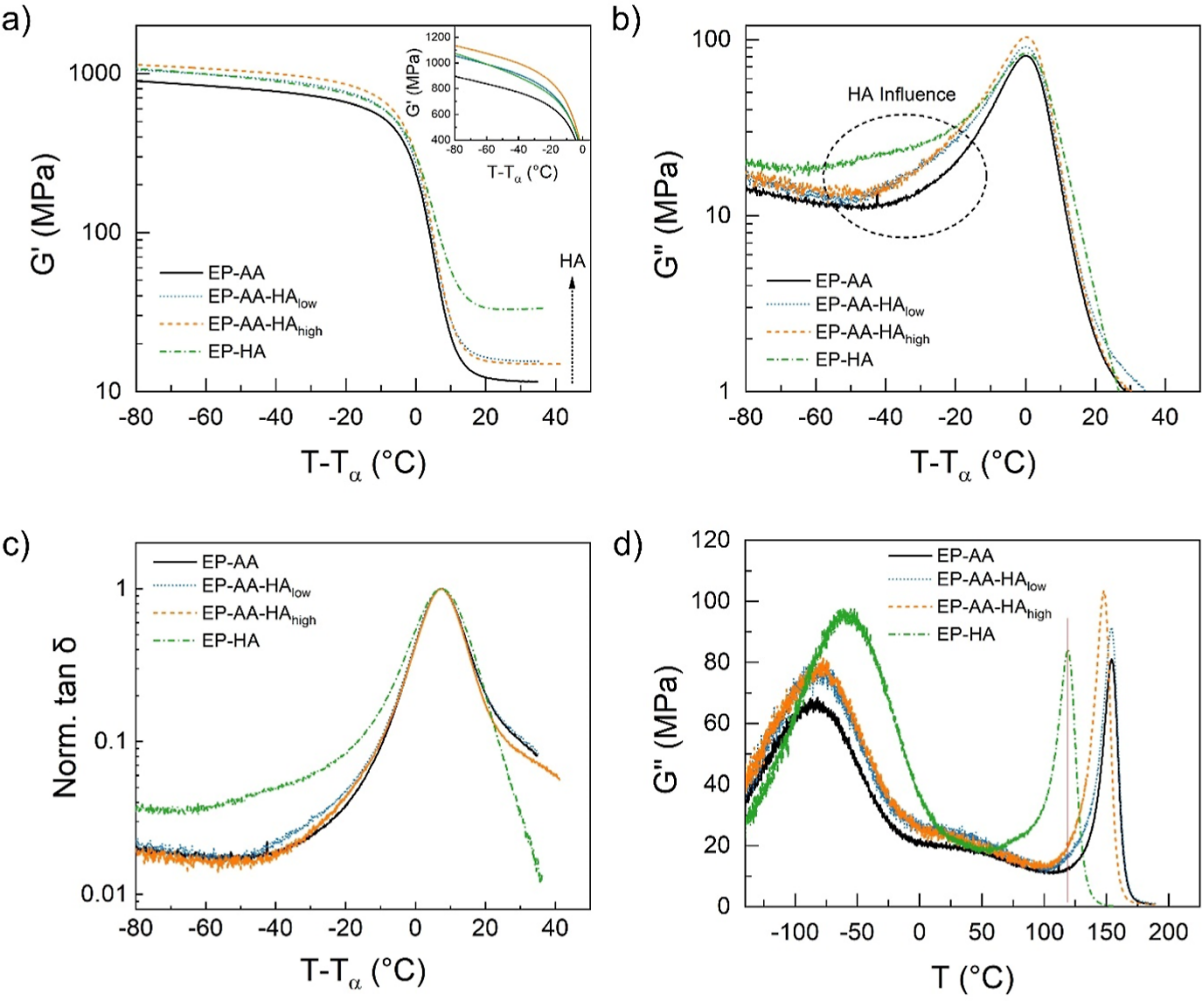
Viscoelastic behavior of epoxy blends, (a) storage modulus (*G*′), (b) loss modulus (*G*″), and (c) normalized loss tangent (tan δ) as a function of temperature at constant frequency (1 Hz); temperature was shifted to *T*–*T*_α_ °C, where *T*_α_ is the α-relaxation temperature. Graph (d) presents the nonshifted temperature data of the specimens’ loss modulus (*G*″) for comparison.

The storage modulus (*G*′) response for all specimens (Figure 4a) shows glassy behavior between 900 MPa and 1150 MPa at 80 °C less than *T*_α_ and rubbery behavior between 10 and 50 MPa at 35 °C greater than *T*_α_. With respect to the α-relaxation process, the *G*′ of the EP-AA control and the HA blend specimens undergo a sharper transition from the glassy to the rubbery state as compared to the EP-HA control, also evident by the breadth of the transition in the *G*″ and tan δ graphs. This behavior can be explained given that the h-PEI has a more heterogeneous structure as compared to the AA, which is attributed to the broadened segmental (α) relaxation process observed in the *G*′ data and is more evident in the *G*″ and tan δ responses, as shown in Figure 4b,c, respectively.^31^

The rubbery moduli (*G*_r_^′^) values across the specimens obtained at around *T*_α_ + 35 °C (as seen in Figure 4a) show an increase based on HA addition, which may be used to experimentally determine the molar mass of the polymer chains between cross-links (*M*_c_) using the theory of rubber elasticity. Although this theory is mainly applied to low cross-link density materials, it is often used as a means of qualitative comparison in epoxies.^44,45^ However, in off-stoichiometric blends containing hyperbranched amines, its validity is highly questionable due to the complex interactions from “non-ideal” or heterogeneous networks. An estimate of the theoretical *M*_c_ values for each composition (denoted as *M*_ct_) is provided in Table 3. The experimentally determined value of *M*_c_ for the EP-AA control (*M*_ce_ = 427 g/mol) is within the commonly observed range for a short, rigid diamine network in a system consisting of a mixture of two trifunctional blocks.^44–46^

However, in the case of the EP-HA control, when the calculations are conducted, assuming an ideal network (φ = 1), and ethylenediamine as the structure of the curing unit (*M*_w_ = 60 g/mol), the theoretically predicted value for *M*_c_ is much larger than the experimentally determined one. This indicates a more highly cross-linked polymer network in the EP-HA specimen, which is to be expected when larger multifunctional amine molecules are used for curing. Prior work from Francos et al.,^29^ examined the differences in two HA systems, one with a molar mass of nominally 800 g/mol and one with a molar mass of nominally 2000 g/mol, and deviations from an ideally cross-linked network were observed in the higher molar mass HA system. This indicates that the 25,000 g/mol HA system that we have examined would be expected to have even greater deviation from ideal behavior than what has previously been observed. This explains our relaxed/rubbery modulus (*E*_r_) value obtained indirectly *G*_r_^′^ of approximately 92 MPa, which is about 1.6 times greater than that observed by Fernández-Francos et al.^31^ It is postulated that the presence of a higher molar mass HA forms tight covalent networks that impose mobility restrictions. These in turn could trap the shorter chains and chain ends, resulting in an increased relaxed/rubbery modulus in the EP-HA specimen. When comparing the experimentally determined relaxed modulus (*G*_re_′) in the blends, it is evident that the addition of HA has a disproportionate effect on these values, as shown in Figure 4 and Table 3. A simple rule of mixture-based calculation, as shown in Table 3, revealed approximately a 25 and 21% deviation between the experimentally determined (*G*_re_^′^) and calculated (*G*_rc_^′^) values in the EP-AA-HA_low_ and EP-AA-HA_high_ blends, respectively. Though a combination of increased cross-link density in the HA network combined with the trapped lower molar mass species and chain ends would suggest an increase in *G*_re_^′^ from the EP-AA to the HA blends, no substantial change was observed between the *G*_re_^′^ values of the EP-AA-HA_low_ and EP-AA-HA_high_ specimens. This may be attributed to the marginally higher offset in the epoxy-amine reaction stoichiometry between the 1 and 0.4% HA blends, which could potentially trap the excess unreacted AA and, in turn, act as a plasticizer for the network. Another possible explanation for this phenomenon could be the effect of increased chain ends from the unreacted secondary amines of the EP-AA network.^39,43,47^ When comparing the glassy moduli (*G*_g_^′^) values obtained from the storage modulus data (Figure 4a) at *T*–*T*_α_ of −80 °C across the specimens, it is observed that the EP-AA control has a modulus of approximately 900 MPa. A subsequent addition of HA at mass fractions (%) with nominal values of 0.4 and 1 is found to increase the glassy moduli of the EP-AA to around 1000 and 1100 MPa, respectively, which may be attributed to the stiffness of the highly cross-linked EP-HA network.

The *G*″ response for all specimens is shown in Figure 4b (nonshifted temperature data in Figure 4d). All the specimens have a single major α-transition peak around 154 °C, however, there is a small shoulder that appears around 125 °C in the blends containing HA. This peak coincides with the major α-transition peak present in the EP-HA sample (at 119 °C, red vertical line in Figure 4d), indicating the possibility of macro- to microphase separation in these specimens.^48^ In addition to the differences in the alpha relaxation temperature (*T*_α_) shown in Table 3, the viscous dissipation by the polymer network during segmental relaxation (as represented by the features of the *G*″ peak) is different between the EP-HA and EP-AA controls. Owing to the differences in the network architecture (as shown in Figure 1) and the molar mass of the amines, the α-relaxation peak in *G*″ of EP-AA is narrower than that of the EP-HA specimen. It has been previously observed that the higher molar mass HA introduces larger internal branching and a more heterogeneous cross-linked network compared to a lower molar mass HA, or an aliphatic amine,^31^ which is similar to that observed here. Furthermore, the EP-HA sample revealed the presence of a second broad peak at around 80 °C or *T*–*T*_α_ of −50 °C (as shown in Figure 4d,b respectively) indicating the possibility of a second relaxation process in this sample, which is also supported by the DSC data in this study revealing the presence of a second α-transition peak. The temperatures for these two peaks in the EP-HA sample from the DMTA study do not match exactly those revealed from the DSC study, especially because the latter was conducted on dynamically cured specimens. However, the delta between these two peaks is approximately 40 °C and is comparable to the one observed from the dynamically cured EP-HA sample during the DSC study (Table 2). Lastly, the blends display an intermediate trend between the EP-AA and the EP-HA control specimens. The experimentally predicted value of *T*_α_ for the EP-AA-HA_low_ blend does not deviate from the EP-AA control, but the addition of HA segments to the EP-AA network causes a minor increase in the *G*″ dissipation peak. This results in the addition of glassy relaxation modes at temperatures between 20 and 40 °C less than *T*_α_, which is approximately in the range of *T*_α_ for the EP-HA specimen. In the case of the EP-AA-HA_high_ blend, there is a further increase in *G*″ peak intensity along with a similar HA influence observed at the glassy region.

The normalized loss tangent responses (tan δ) are shown in Figure 4c to highlight the breadth of the segmental relaxation process. Since tan δ is defined as the viscous dissipative properties of the matrix compared to its elastic response (i.e., *G*″/*G*′), the area under the tan δ plot is often used as an indicator of energy dissipative properties of the polymer matrix. However, it must be noted that responses at different time and temperature conditions need to be developed to fully describe the area under the tan δ curve.^28^ As the area under the curve is difficult to estimate from the current study, only a qualitative comparison is presented. The EP-HA has increased relaxation contribution in the glassy region, between 40 and 80 °C, and lower contributions in the rubbery region, between 20 and 40 °C, compared to the EP-AA specimen, which may be a consequence of heterogeneous, lower *M*_w_ branches in the HA architecture. In the case of tan δ, the blends follow a trend that is closer to the EP-AA specimen, as indicated by the less pronounced glassy relaxation modes less than the *T*_α_ compared to the trends observed for the *G*″ (Figure 4b). This indicates that there is a similar increase in the *G*′ and the *G*″ responses; therefore, the overall area under the tan δ response for the HA blends remains unaffected. Though there is a slight increase in the breadth of the glassy relaxation contributions in the EP-AA-HA_low_ blend, this behavior is not observed in the higher HA composition blend (EP-AA-HA_high_). Furthermore, there is a reduction in the rubbery contribution for the EP-AA-HA_high_ blend and likely a reduction in the area of the tan δ peak as compared with the EP-AA control. Overall, the HA blends here are not expected to display improved toughness when compared to the EP-AA control.

The *T*_α_ values of all isothermally cured specimens determined from the peak of *G*″ is shown in Table 3, and the *T*_g_ values determined by DSC (*T*_ge_) at a heating rate of 10 °C/min are also included for comparison. The DMTA measurements present the temperature at which the majority of the polymer backbone undergoes transition based on oscillatory mechanical loading at a particular frequency. In contrast, the DSC technique relies on the changes in the heat capacity of a relatively small sample mass while undergoing these transitions. Due to these fundamental differences, the *T*_α_ from DMTA and the *T*_g_ from DSC are not expected to be similar. However, these numbers are in good agreement (Table 3), as observed previously on composites based on a similar resin system, with a less than 10% difference.^28^ The *T*_g_ measurements of the EP-AA and EP-HA controls are expected to be mostly homogeneous and have low standard deviations. However, due to the heterogeneity in the polymer network introduced by HA, the two blends studied here display larger variations in *T*_g_ across samples derived from a single processing condition compared to the control specimens.

A direct comparison between *T*_g_ values determined from isothermally cured samples versus the dynamic curing experiments (Table 2) is difficult to make because of the differences in the heating rates and the thermal history of the specimens used in these experiments. However, it is acknowledged that dynamic curing experiments often suffer from diffusion limitations,^14,49^ while the DMTA specimens often suffer from minor surface oxidation due to long measurement durations, both of which may contribute to the 5% difference in *T*_g_ values observed between the dynamically cured and isothermally cured AA control and the HA blends. However, a higher than expected difference (approximately 19%) in *T*_g_ is observed in the EP-HA control between specimens cured dynamically in the DSC and isothermally cured in a convection oven. In addition to the differences in the *T*_g_ values, the shape of the glass transition curve for the dynamically cured EP-HA sample indicated the presence of a second *T*_g_, as previously discussed, and the shape of the thermogram is broad and occurs over a range between 50 and 140 °C, while it is narrower (occurring between 80 and 140 °C) in the oven-cured counterparts. A broader transition is indicative of a heterogeneous network and has been observed in copolymer blocks and blends based on interactions between different molar mass species.^50^ Finally, upon the examination of the similar values of the Δ*H* across the dynamic cure experiments (as shown in Table 2), it is hypothesized that the HA diffusivity and the reactivity of the inner amine hydrogens may be affected by the heating rate. The network structure in the EP-HA specimen is expected to differ based on the heating rate conditions and the cure temperature.^29,33^

The β-transition temperatures (*T*_β_) estimated from the peak of the *G*″ response at temperatures less than the segmental relaxation temperature (Table 3), and the *G*″ response against the normalized temperature greater than *T*_β_ for each specimen are shown in Figure 5. The β-transition process is attributed to cooperative local mobility within cross-linked networks,^51^ typically arising from side group motion.^52^ The *T*_β_ shown in Table 3 agrees with the trend that increasing HA concentrations increases the cross-linking density (1/*M*_c_) within the specimens, which reduces the cooperative side chain mobility within these specimens, as observed previously.^44,52^ However, due to the addition of end groups from the hyperbranched structure of h-PEI, the intensity of these processes is found to increase as a function of the HA concentration, as shown in Figure 5. Although, correlations between side chain mobility and toughness have been observed in literature,^52^ the presence of excess amine in the blends may lead to heterogeneous interactions and increased chain ends. This contributes to the increased side chain mobility observed here and does not contribute to improved energy dissipative characteristics in the HA based blends, as discussed later.

**Figure 5:**
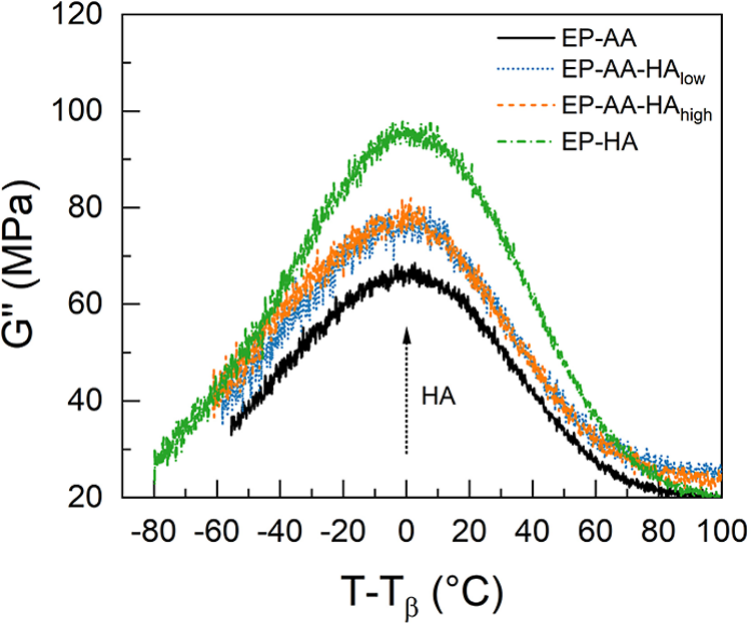
Loss modulus (*G*″) as a function of *T*–*T*_β_, showcasing the β-transition process at temperatures less than the segmental relaxation.

### 3.4 Nanoscale Porosity and Free Volume

The nanoscale structural porosity, or free volume, of polymers corresponds to the inter- and intramolecular vacancies between polymer chains and has been shown to affect macroscale properties such as viscoelastic characteristics, glass-transition, density, and material toughness. Positrons have been shown to be a powerful probe to evaluate this because their lifetime in condensed matter is a direct function of the electron density within the material.^53–56^ In the case of polymers, the lifetime of the orthopositronium (o-Ps) (a metastable state of a positron and an electron) “pick-off” process is dependent on the electron density of the free volume sites.^57,58^ In the absence of chemical interference between positronium and chemical moieties within polymers, the variations in the o-Ps lifetime (τ_o‑Ps_) and the corresponding number density of o-Ps (intensity, *I*_3_) are used as metrics to compare free-volume characteristics (average nanoscale porosity) across specimens.^57–59^

Though PALS-based analyses are widely used to describe macroscale trends in composites, blends, etc.,^60^ the analysis aspect is nontrivial when examining small, incremental changes in composition such as those considered here. In our previous study, PALS was successfully utilized to differentiate structural characteristics between plain epoxy and fiber composite systems using a simple 3-lifetime, nonlinear least-squares-based exponential fitting process.^28^ Here, a similar approach was adopted for a preliminary analysis, and the outcomes of PALS measurements under room-temperature conditions and atmospheric pressure are shown in Table 4.

**Table 4:** Orthopositronium “Pick-Off” Lifetimes (τ_o‑Ps_) with Average Nanoscale Pore Size Values (*R*), % Number Density of Pore Sizes, and the Fitting Quality Parameter (*X* ^2^) for the Various Samples Used in the Study

sample	τ_o‑Ps_ (ns)	*R* (Å)	*I*_3_ (%)	reduced *X* ^2^
EP-AA	1.659 ± 0.003	2.544 ± 0.003	19.25 ± 0.06	0.98 ± 0.02
EP-AA-HA_low_	1.686 ± 0.004	2.573 ± 0.004	20.70 ± 0.08	1.02 ± 0.02
EP-AA-HA_high_	1.691 ± 0.005	2.591 ± 0.005	19.99 ± 0.11	1.02 ± 0.02
EP-HA	1.503 ± 0.004	2.363 ± 0.005	17.71 ± 0.10	1.01 ± 0.02

Comparing the average (τ_o‑Ps_) and the corresponding average pore size (R) calculated using the Tao-Eldrup model^61^ between the EP-AA and the EP-HA specimens, it is evident that the aromatic structure (AA) introduces larger free volume sites than the hyperbranched architecture (HA). Further, it was also observed that the number density of o-Ps annihilation sites in the EP-HA is relatively lower than in the EP-AA specimen, indicating fewer free volume sites and likely a denser EP-HA structure. Upon comparison of the HA-based blends, it is evident that the addition of HA is found to increase the average nanoscale porosity or the free-volume sites in these materials, similar to that observed in our previous study, where the average nanoscale porosity in the fiber composites was found to increase upon addition of h-PEI functionalized CNT. Further, studies in HBP-based blends indicate increases in free volume in micro- and nanoscale phase-separated blend systems that is similar to the response observed here.^48,62^

Based on the preliminary trends shown in Table 4, a more rigorous analysis methodology was adopted. Though average exponential lifetimes are useful for ascertaining trends, it has been demonstrated that (τ_o‑Ps_) in polymers often follow a distribution owing to the heterogeneity in polymer networks.^60,63,64^ Therefore, a modified NLLS technique is used where it is assumed that exponential decay of (τ_o‑Ps_) is broadened by a log-normally contributed distribution of lifetimes,^65^ as shown in Figure 6. The average (τ_o‑Ps_) obtained from this analysis is slightly less than that shown in Table 4. Typically, the centers of mass of the lifetimes (average values) are adjusted in the fitting process when a distribution is assumed in place of discrete exponential values. Overall, the trends across the lifetimes are similar to Table 4. The addition of HA to EP-AA increases the average lifetime in the blend specimens compared to that of the EP-AA control, as shown in Figure 6a. The notable aspect of this analysis is the broadening (σ_o‑Ps_) or heterogeneity of the network presented in Figure 6b. Overall, the EP-AA sample has a larger distribution of lifetimes (≈0.25 ns) as compared to the EP-HA control (≈0.15 ns), despite the presence of chain ends and branch nodes in the EP-HA network compared to the EP-AA network. At 0.4% HA concentration, there is a slight decrease in the heterogeneity; at 1% HA concentration, there is a more prominent increase in the heterogeneity of the blend specimen. Owing to the large standard deviations observed in Figure 6b, these trends are also verified using a continuous analysis methodology.^66^

**Figure 6:**
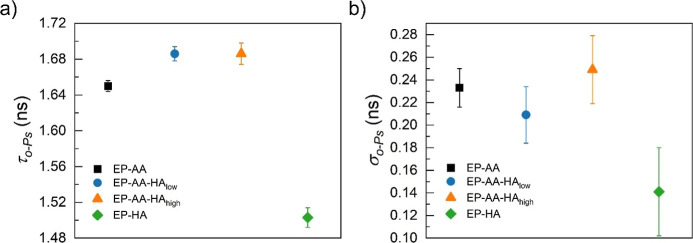
Positron annihilation spectroscopy data, (a) lifetime of orthopositronium (τ _o‑Ps_), and (b) broadening of the network (σ_o‑Ps_) along with the corresponding distributions in the specimens. Data presented are mean ± standard error of the mean (S.E.M.), with *n* ≥ 3.

Although EP-HA presents lower (τ_o‑Ps_) and (σ_o‑Ps_) values than the EP-AA specimen, the addition of HA to EP-HA does not follow a decreasing trend. This suggests that the HA curing in the blends introduces a different network architecture from the EP-HA control. The structural incompatibility and the *M*_w_ differences between the amines (HA and AA) may introduce minor phase separations (as shown in Figure 4b,c). However, the presence of excess amine mass fractions also introduces unreacted amine chain ends in the blends (as previously discussed in this manuscript) that may result in an increase in average nanoscale porosity and network heterogeneity in these materials.^67^ Though the relationship between free-volume and *T*_g_ of materials for unentangled polymers is well established using the Flory–Fox equation shown in eq 6 below,^68^ it is often applied toward cross-linked materials to provide a qualitative comparison.

6 $$T_{g}-T_{g\infty }=\frac{K}{M_{c}}$$

where *T*_g∞_ is the *T*_g_ for a given material at the highest-cross-linked state (lowest *M*_c_) possible. According to eq 6, the *T*_g_ of a given material is directly proportional to the free volume-based material parameter (*K*) and inversely proportional to the *M*_c_. It is noted that PALS measurements in the current study are conducted only at a single temperature, and the relative changes in free volume as a function of temperature are necessary for modeling *T*_g_ behavior in materials.^53,69^ However, a likely scenario is postulated here. Equation 6 predicts that a decrease in *M*_c_ (or increase in cross-link density) will cause an increase in *T*_g_ for a given material structure. However, in the current study, the addition of HA to the EP-AA blends also increases side group contributions from the large number of primary and secondary amines present (as shown in Figure 5). It is expected that the increased branching in HA architecture and the presence of excess unreacted amines increase the overall chain end contributions within the blend specimens that plasticize the network and lead to poor polymer packing and an increase in the nanoscale free volume. This increase in free volume may result in the *T*_g_ decrease observed in the blend specimens.

### 3.5 Tensile Testing

The polymer samples were cast into dog-bone specimens for mechanical testing to evaluate the effect of HA addition on the tensile properties of the blends. The representative stress (σ) vs strain (ε) plots and the summary of tensile tests carried out on dogbone samples made from the various specimens are shown in Figure 7 and Table 5.

**Figure 7:**
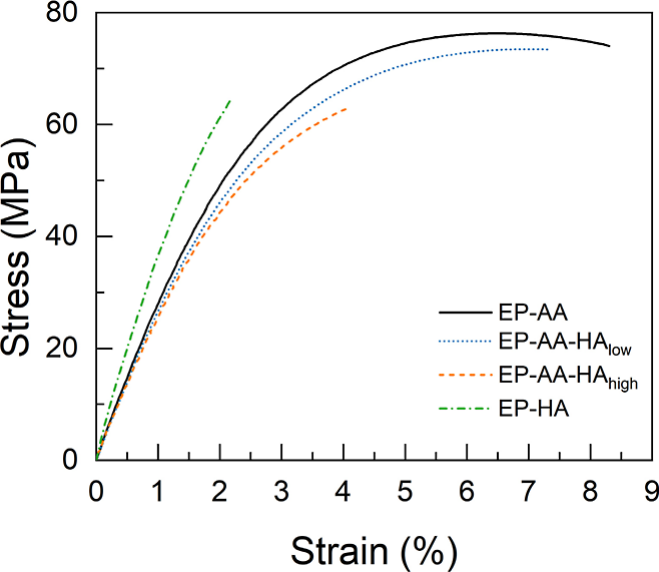
Representative stress vs strain curves for the different isothermally cured epoxy blends used in this study; data collected at room temperature.

**Table 5:** Tensile Test Results and Values of Mechanical Properties for Specimens Used in the Study

sample	UTS (MPa)	*E* (GPa)	ε_f_ (%)
EP-AA	75.7 ± 2.0	3.1 ± 0.4	9.0 ± 1.2
EP-AA-HA_low_	73.4 ± 2.1	2.8 ± 0.1	7.1 ± 1.4
EP-AA-HA_high_	57.7 ± 3.7	2.6 ± 0.2	3.5 ± 0.5
EP-HA	61.5 ± 2.0	3.4 ± 0.5	2.4 ± 0.3

From Figure 7, it is evident that the control samples, EP-AA and EP-HA, show different stress–strain behaviors. EP-AA specimens display a more ductile response and have higher UTS and strain to failure (ε_f_) than EP-HA specimens, which predominantly display a brittle response along with a higher overall stiffness (Young’s modulus of elasticity (*E*)) than EP-AA. In general, UTS is a direct function of the strength of the polymer backbone, and ε_f_ is directly correlated with the ability of the polymer network to rearrange and dissipate the energy applied during mechanical loading. The trends in mechanical data are in accordance with and can be explained using previous observations from the other techniques used in this study. It is well-known that aliphatic structures display lower strength than aromatic structures, and this is also confirmed in the current study where the UTS of EP-AA is higher than EP-HA.^32,70^ From Table 3, it is evident that EP-AA specimens have higher *M*_c_ (lower cross-link density) than EP-HA and, therefore, form a less dense polymer network with larger nanoscale porosity (Table 4) that provides conformational freedom for higher polymer chain mobility in these materials. This may allow for better energy dissipative behavior, thereby increasing their ductility and strain to failure compared to EP-HA. Further, the reduction in average nanoscale porosity and the reduced free-volume sites may be correlated to an increase in specimen density, as previously observed by Denis et al.,^55^ that may lead to moduli improvements observed in the EP-HA specimens compared to EP-AA.

The property changes observed in the blends are more complex compared with the control specimens. The addition of 0.4% HA (EP-AA-HA_low_) only causes minor reductions in mechanical properties, especially the strain to failure, as seen in Table 5, but the overall mechanical response is still similar to EP-AA. However, at 1% HA concentration (EP-AA-HA_high_), the response of the matrix changes from ductile to primarily brittle, and reductions of 25% drop in UTS and approximately 61% in ε_f_ are observed. As shown in Table 5, UTS of EP-AA and EP-HA present the extremes in mechanical behavior where EP-AA is ductile and EP-HA is brittle. If favorable network interactions between EP-HA and EP-AA exist, the 1% HA blend is expected to have a nominal loss in strength that is more in accordance with that observed in the 0.4% HA composition. However, the unusual loss in UTS cannot be explained just based on the presence of increased aliphatic functionality. It is hypothesized that during the curing process, the reaction between primary aliphatic amines of HA and the EP autocatalyze the curing process of the primary aromatic amines and EP, thereby forming cocontinuous, heterogeneous network phases with possible microscale phase separations due to *M*_w_ differences between AA and HA. Postgelation, the diffusion barrier to the secondary HA amine sites is increased, as observed from the increased *E*_a_ for the cure reactions (Figure 3), and the secondary AA is preferentially cured, thereby increasing the presence of unreacted secondary aliphatic amine and tertiary amine chain sites. Though the overall cross-link density of the polymer blends is increased from the addition of HA, at 1% HA concentration, the cross-link density is comparable to that of the 0.4% HA specimens. This indicates the possibility of greater defective sites due to unreacted amine groups in the network that lead to poor transfer between the aromatic and hyperbranched networks and thereby reduce the UTS of the 1% HA composition. Moreover, the increase in chain ends also increases network heterogeneity and packing frustration, leading to increased free volume but poor energy dissipative properties in the 1% HA blend, as evidenced by the decrease in ε_f_.^67^

### 3.6 Fractography and Structural Morphology

Cross-sectional areas from the failure points of the tensile tested specimens are examined using an SEM. Regardless of the highly cross-linked network architecture of the specimens, the majority of the samples (except EP-HA) exhibit both brittle and ductile failure domains. However, energy dissipative mechanisms and sample ductility are characterized by surface roughness of the fractured surfaces (Figure 8), where a rougher surface is indicative of greater resistance to failure compared to a relatively smooth surface.^67,71^ Based on this, it is evident that the surface roughness observed in EP-AA and EP-AA-HA_low_ (Figure 8a,b) are indicative of their superior strain to failures compared to EP-AA-HA_high_ and EP-HA (Figure 8c,d). Qualitatively, the feature sizes in EP-AA and 0.4% HA composition are comparable and are in the 100s of μm range, and these compositions exhibit nearly similar ε_f_ (Table 5). It is interesting to note that despite only a nominal increase in HA composition from 0.4 to 1%, the fracture cross-section of the 1% HA composition is drastically different compared to the 0.4% HA specimen. As observed in Figure 8c, there are fewer surface features (tears) that are sub 100 μm in size range that likely provide lower resistance to failure propagation in 1% HA composition. Finally, as is evident from the low ε_f_ values (Table 5), the EP-HA exhibits a relatively smooth fracture surface that is indicative of minimal resistance to failure.

**Figure 8:**
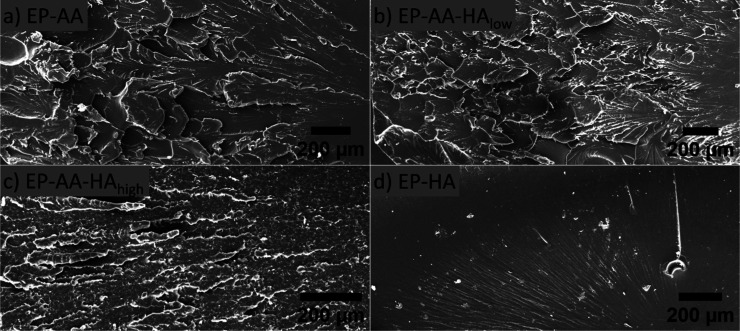
Surface morphology of cross-sectional areas from the failure regions of the different epoxy blends post tensile testing.

High-magnification SEM images of the failure regions corresponding to Figure 8 are shown in Figure 9. At this magnification, the focus is shifted toward observing possible phases corresponding to the HA and AA networks, respectively. Overall, EP-AA does not display any particular structural domains or specific failure characteristics owing to its relatively homogeneous structural composition compared to the blends. At 0.4% HA composition, structural domains start to emerge, ranging between 50 and 100 μm in size (highlighted in blue). These domains repeat across the fractured surface of this epoxy blend and show very similar architecture and characteristics. The center of these domains appears to be uniform, which is typical of brittle failure responses, with tears at the edges (brighter edges protruding out of plane) due to resistance to failure. At 1% HA composition, it is observed that the sizes of domains reduce to tenths of μm (some representative areas are shown in yellow), which is likely an indicator of the increased contribution of HA to the overall network architecture. These features can be observed across the entire surface of the fractured sample in this higher magnification image, and they look like “tiles” with white edges along their perimeter due to increased resistance to failure around the edge of each domain. The SEM images do not indicate typical phase separation behavior as observed previously^11,48^ and as confirmed by the optical micrographs no changes in optical transparency were observed across the blends. However, the failed surfaces do indicate the formation of cocontinuous domains whose size ranges seem to decrease as a function of HA percentage. Based on this trend, it may be likely that the addition of higher HA percentages may introduce immiscibility due to increased viscosity differences between EP-HA and EP-AA mixtures^11,48^ and introduce possible phase separation during the curing process consisting of smaller particle sizes (near μm size range) that may relieve stress concentrations in the blends and improve toughness, as observed previously.^48^

**Figure 9:**
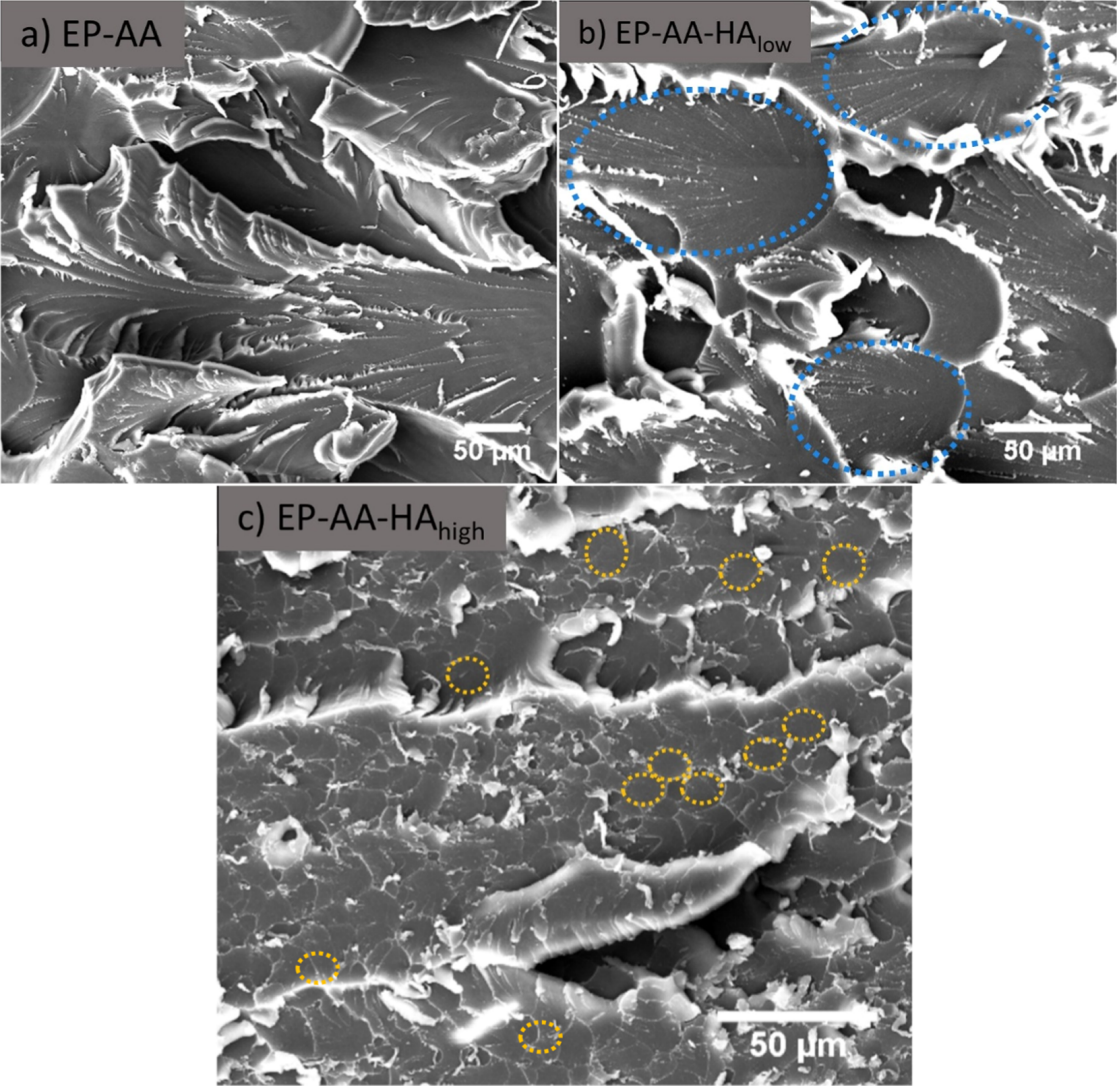
High-magnification images of failure regions for the different epoxy blends studied.

## 4 Conclusions

This study investigates the effects of adding h-PEI (HA), a HBP, on the overall properties of an epoxy matrix in the absence of elastic reinforcements, such as fibers and nanoparticles. We evaluated the curing process and studied the structure–properties relationship. The results of this study demonstrated that the curing of commercial epoxy materials is significantly affected by the presence of larger molar mass hyperbranched amine molecules in the blend. Differences in the cure kinetics, structure, thermal, and mechanical properties were observed between control samples (EP-AA) cured with low molar mass aromatic amines and their hyperbranched amine cured counterparts (EP-HA), as well as blends of the above at two different weight percent compositions, namely, 0.4% HA (EP-AA-HA_low_) and 1% HA (EP-AA-HA_high_), respectively. In addition, subtle differences were observed in the thermomechanical properties between the dynamically and isothermally cured samples. The results of this study may indicate that the addition of larger amounts of HA in the epoxy composition can result in phase separation during the curing process and formation of semicontinuous domains whose size decreases as the amounts of HA increase. This is a plausible outcome of the diffusion limitation issue of the larger molar mass and branched architecture of HA molecules compared to the significantly smaller AA molecules used in the control material. This leads to increased viscosity during the curing process and the formation of tight covalent networks that impose mobility restrictions. These topological constraints, as the curing process takes place over time, can create domains within the material with higher concentrations of epoxy and others with higher concentrations of HA, resulting in lower amine reactivity and possibly an incomplete cure, as suggested by the lower exothermic heat of reaction in the EP-HA specimen. Ultimately, the data collected indicate that the addition of higher HA percentages may introduce immiscibility due to increased viscosity differences between EP-HA and EP-AA specimens and introduce possible phase separation during the curing process consisting of smaller particle sizes that may relieve stress concentrations in the blends and improve toughness while reducing the brittleness of these materials. These findings may help advance the field of epoxy cured materials used in various applications and especially the field of epoxy reinforced fiber composites.

## Data Availability

The data that support the findings of this study are available on request from the corresponding author.

## Footnotes

a The full description of the procedures used in this paper requires the identification of certain commercial products and their suppliers. The inclusion of such information should in no way be construed as indicating that such products or suppliers are endorsed by NIST or are recommended by NIST or that they are necessarily the best materials, instruments, software, or suppliers for the purposes described.
